# Editorial: Pediatric Critical Care in Resource-Limited Settings

**DOI:** 10.3389/fped.2019.00080

**Published:** 2019-03-20

**Authors:** Srinivas Murthy, Krishan Chugh, Ndidi Musa, Yves Ouellette, Phuc H. Phan

**Affiliations:** ^1^Department of Pediatrics, University of British Columbia, Vancouver, BC, Canada; ^2^Fortis Healthcare, New Delhi, India; ^3^Department of Pediatrics, University of Washington, Seattle, WA, United States; ^4^Mayo Clinic, Rochester, MN, United States; ^5^Medical Intensive Care Unit, Vietnam National Children's Hospital, Hanoi, Vietnam

**Keywords:** critical care, pediatrics, global health, health equity, research agenda-setting, health workforce

The young field of pediatric critical care has reached an inflection point in its growth. Over its first few decades, practice was driven from a select few intensive care units in rich countries that produced the bulk of our fields' research and served as the training ground for its practitioners (1). The past 10 years, however, have revolutionized our field. There are pediatric critical care units in nearly every country in the world that provide life-saving care to more children than at any point in history (2).

Alongside that growth, we have realized that the research we perform needs to reflect the needs of those children. Given the hugely discrepant global burden of pediatric mortality, this means ensuring that children in lower-income regions of the world are kept at the forefront of any research agenda. As Paul Farmer of Partners in Health has stated, achieving excellence without achieving equity is the main human rights dilemma of healthcare in the twenty-first century, and no field is more emblematic of that challenge than pediatric critical care.

In this issue of Frontiers in Pediatrics, we have collected a diverse array of manuscripts examining a variety of issues pertaining to the critically ill child in lower-income countries. The recent growth in the field brings with it new challenges that deserve thoughtful consideration if our field is to grow in an evidence-driven, cost-effective, and ethical way.

## A Well-Trained Workforce

As many have stated, maintaining a well-trained health workforce is likely the biggest challenge in achieving a higher standard of care for those around the world (3). A lack of training opportunities and the ongoing brain-drain leads to a vacuum of skilled clinicians to provide advanced pediatric care in many regions of the world.

Collaboration for education is described, focusing on ensuring that materials are relevant to local practitioners. Primarily, focusing any educational opportunities on what communities want through needs assessments allows teachers to incorporate the varied learning styles and needs around the world with what is practical to implement. As care is often provided by those with limited training in critical care, teaching the fundamental principles to non-intensive care physicians can be achieved by adapting standardized short term pediatric critical care training courses to fill in the gaps. Ultimately, establishing formal training programs that are locally-organized with locally-driven rules and local accountability will be the path to ensuring a sustainable workforce. Practitioners from high-income countries must support these programs at all possible opportunities, facilitating local control of training opportunities to foster sustainability.

## Understanding the Burden of Pediatric Critical Illness

A major part of targeting research to need is in defining an agenda. Fundamental to that is ensuring that research performance has the appropriate infrastructure in place to be done well. Establishing regional registries has been incredibly valuable in many high-income regions, and similar experiences are being rolled out in lower-income countries, such as India and Sri Lanka. Yet, challenges remain in performing well-designed research in much of the world, especially in designing and performing well-crafted implementation programs that are vital to translate research knowledge into practice.

Specific research will be highlighted to demonstrate how region- and population-specific epidemiology can be targeted to improve our understanding of disease and impacting outcomes. The burden and impact of specific viral infections vary by geography and socio-economic status. The management of fungal infections is unique in some regions, compared to others. Trauma management in resource-limited systems requires a drastically different approach from what many in high-income countries are familiar. Sepsis management, as always, will be context and resources-dependent. Most crucially, palliative care, which is an often under-appreciated component of critical care practice, deserves much more attention in the global literature. As demonstrated from well-known studies such as FEAST (4), for research to be relevant it must involve participants that are from the affected communities and reflect local practice and disease patterns.

## The Ethics of Cost-Intensive Care

From a practical standpoint, the ethics and economics of providing resource-intensive care when resources are constrained needs to be adequately explored. Simply because we *can* provide interventions does not necessarily mean that we *should* provide those interventions, both at the individual and the health system level. Balancing effective public health interventions such as vaccination and ensuring access to primary care with the resource-intensity of critical care requires a focused understanding of the principles of distributive justice and resource allocation. Assessing the appropriateness of this high-cost care should be an integral part of the growth of our field, not just in low-income regions, but in all regions around the world as the technological complexity of the care we provide continues to increase.

The history of pediatric critical care is a short one, and researchers in lower-income countries have begun to ensure that they will be at the forefront of future advances. Innovations and research from lower-income regions are informing practice globally, with a number of high-impact randomized trials changing how the world manages sick children (4–7). We are slowly beginning to match the burden and distribution of disease with research to improve outcomes from those diseases, and are breaking out of the antiquated top-down approach of knowledge dissemination. This issue of Frontiers in Pediatrics is hopefully another step in that direction.

As high-income countries learn from their low-income country colleagues, working toward improving the resources available and the determinants of pediatric critical illness must be a collective ambition. As pediatric critical care providers in both higher- and lower-income regions, we call on our field to unite behind this common goal of improving the still-too-high rates of child mortality around the world. Through high-quality, patient-focused research dedicated on improving outcomes in populations most at-risk, we can achieve both excellence and equity.

## Author Contributions

All authors listed have made a substantial, direct and intellectual contribution to the work, and approved it for publication.

### Conflict of Interest Statement

The authors declare that the research was conducted in the absence of any commercial or financial relationships that could be construed as a potential conflict of interest.
